# Bioactive Substances, Heavy Metals, and Antioxidant Activity in Whole Fruit, Peel, and Pulp of Citrus Fruits

**DOI:** 10.1155/2021/6662259

**Published:** 2021-03-16

**Authors:** Anna Czech, Agnieszka Malik, Bożena Sosnowska, Piotr Domaradzki

**Affiliations:** ^1^Department of Biochemistry and Toxicology, Faculty of Biology and Animal Production, University of Life Sciences in Lublin, Akademicka 13, 20-950 Lublin, Poland; ^2^Department of Biotechnology, Microbiology, and Human Nutrition, Faculty of Food Science and Biotechnology, University of Life Sciences in Lublin, Skromna 8, 20-704 Lublin, Poland; ^3^Department of Commodity Science and Processing of Raw Animal Materials, Faculty of Animal Sciences and Bioeconomy, University of Life Sciences in Lublin, 20-950 Lublin, Poland

## Abstract

The use of whole citrus fruits in the food industry means that the valuable peel is used, but this may raise palatability or health concerns among consumers. The content of sugars, dietary fibre, redox compounds, lead, and cadmium was compared in citrus fruits (orange; pomelo; mandarin; lemon; key lime; and red, yellow, and green grapefruit). The pulp of all fruits contained significantly less fibre, tannins, and phenolic compounds than the peel. Whole citrus fruits had significantly lower content of sugars and higher content of dietary fibre and phenolic compounds, including ferulic acid, than their pulps. Whole grapefruits had higher concentrations of ascorbic acid. Whole lemons, limes, and mandarins had higher antioxidant potential than their pulp, due to their higher content of ascorbic acid, tannins, and phenolic compounds. Lead and cadmium content in whole fruits, while higher than in the pulps, was well below the acceptable daily intake.

## 1. Introduction

Citrus fruits (the family *Rutaceae*) exert health-beneficial effects by stimulating the immune, cardiovascular, and digestive systems. They also have anti-inflammatory, antiatherosclerotic, antibacterial, and anticancer properties [1, 2]. These properties result in part from the presence of numerous bioactive compounds, such as ascorbic acid, tocopherols, carotenoids, dietary fibre, minerals, and a number of other compounds, such as flavonoids, phenolic acids, and tannins, which can and should be an integral part of a balanced diet [3, 4]. Most of these compounds have antioxidant properties that coordinate and stimulate metabolic transformations to protect tissues and body fluids from damages associated with the presence of reactive oxygen species [5].

The amounts and types of bioactive compounds and their antioxidant power are highly diverse and depend on the species of fruits, its variety, or the part of the fruit, as well as on climatic and cultivation conditions [6]. Studies have shown that in most fruits, a significant proportion of the substances showing biological activity are found in the peel, and not in the commonly consumed pulp. Examples include the peels of citrus fruits, apples, grapes, and berries, whose peel is the main source of natural antioxidants [7–9].

In addition to valuable bioactive substances, the peel is also a very valuable and underappreciated source of dietary fibre, which slows down and reduces the absorption of sugars from fruit [2, 10]. Therefore, it would be highly beneficial to use whole citrus fruits in processing, e.g., for jams, juices, mousses, and nectars. However, despite its high nutritional value, the peel is usually dried, mixed with dried pulp, and used as cattle feed, or treated as waste, which can constitute as much as 50% of the original weight of the fruit [11, 12]. This is due in part to concern that the peel may be a source of xenobiotic substances, including heavy metals [13], as well as its bitter flavour. However, some consumers accept this flavour and even seek out such products. The peel of citrus fruits is used not only as a flavouring in the form of spice mixtures or syrups or as a cake ingredient, but also as a separate product (e.g., chocolate-covered or candied peel). Orange peels are also the basis for certain beverages or liquors, in which a bitter flavour is desirable to consumers. Therefore, the bitter taste of the peel would not entirely eliminate the use of whole fruits.

With the rise in the popularity of bioactive compounds and the concept of functional food, food products enriched with citrus peel have begun to appear. In order to take full advantage of the benefits of citrus fruits, it is very important to analyse their composition. Knowledge of the chemical composition of the citrus peel, pulp, and whole fruit may encourage their use in the food and pharmaceutical industries, hence the need for this type of research. It is also interesting to compare citrus fruit varieties popular on the European market in terms of the health-promoting potential (e.g., dietary properties and antioxidant capacity) of their individual parts, i.e., peel *vs*. pulp and pulp *vs*. whole fruit.

There are many studies on the content of bioactive substances in the pulp, peel, or juice of citrus fruits, but their scope is very narrow. For example, Ghasemia et al. [14] presented a study on antioxidant capacity, i.e., total content of phenols and flavonoids and DPPH value, but only in the peel of citrus fruits. Analyses of the content of phenolic acids in the peel can be found in studies by Gorinstein et al. [15] and Elkhatim et al. [3]. Rehman [16] also analysed the antioxidant content of the peel of citrus fruits. Matsuo et al. [17] tested the content of AA, KT, and dietary fibre in the peel of *Citrus natsudaidai*. Chau and Huang [18], Figuerola et al. [19], and Rafiq et al. [2] performed analyses of the content of dietary fibre and its fractions in the peel alone. In contrast with our study, these authors focused mainly on comparing the content of a few bioactive substances, usually in one or two varieties of citrus fruit. There is a lack of research comparing the content of nutrients, antioxidants, and xenobiotics (heavy metals) between the peel, pulp, and whole fruits. These questions are dealt with in our work, which unquestionably enables a broader and more comprehensive view of the nutritional properties of citrus fruits. This is particularly important in planning diets and can be helpful in using specific fruits in industry.

Therefore, the aim of the study was to compare the content of sugars, dietary fibre, phenolic compounds, ascorbic acid, and tannins, as well as the antioxidant capacity (DPPH^•^ and ABTS^•+^) of eight species and cultivars of citrus fruit, i.e., orange (*Citrus sinensis*); pomelo (*Citrus maxima*); mandarin (*Citrus reticulata* Blanco); lemon (*Citrus limon*); key lime (*Citrus aurantifolia*); and red, yellow, or green grapefruit (*Citrus paradisi*), available on the Polish market and originating in the Aegean region in Turkey. The study also analysed whether whole fruits are a source of excessive intake of the heavy metals (cadmium and lead) by potential consumers. The results can be treated as a guide for consumers on the use of whole citrus fruits or their pulp or peel.

## 2. Material and Methods

### 2.1. Plant Materials

The research material consisted of fresh citrus fruits belonging to the genus *Citrus* L. in the family *Rutaceae*. The following species of citrus fruit were analysed:

- Orange (*Citrus sinensis*), Navelina cultivar (Turkey)
- Mandarin (*Citrus reticulata* Blanco), Clementina cultivar (Turkey)
- Lemon (*Citrus limon*), Interdonato cultivar (Turkey)
- Key lime (*Citrus aurantifolia*), Tahiti cultivar (Turkey)
- Pomelo (*Citrus maxima*), Honey cultivar (Israel—the only country supplying this fruit to the Polish market)
- Red grapefruit (*Citrus paradisi*), Star Ruby cultivar (Turkey)
- Yellow grapefruit (*Citrus paradisi*), Duncan cultivar (Turkey)
- Green grapefruit (*Citrus paradisi*), Sweetie cultivar (Turkey)

All fruits were purchased at a supermarket in Poland at the same time in 2019.

### 2.2. Preparation and Mineralization of Samples

Primary samples of each fruit were taken from three different packages from one supplier. Three fruits were taken randomly from each package. In total, 72 pieces of fruit were used for the research (3 fruits × 3 packages × 8 species).

To prepare laboratory samples, each fruit was cut in half and one half was homogenized, treating the sample as a whole (peel + pulp), while the other half was peeled and the pulp (F) and peel (P) were homogenized separately. These steps were performed immediately after purchase. Prior to homogenization, each fruit was washed separately under water (about 60–70°C) and dried with a paper towel to remove impurities that could affect the assay result. The samples were homogenized using the BUCHI mixer B-400 with ceramic blades. The homogenized samples were placed in plastic flasks and kept deep-frozen at -80°C until analysis.

The homogenates were freeze-dried for the determination of sugars, total phenolic content, phenolic acids, and total antioxidant activity using the DPPH and ABTS radicals.

### 2.3. Chemicals and Solutions

Dietary value was analysed according to the AOAC Official Method 992.16 for total dietary fibre [20].

Lyophilisates were diluted with water (1 : 9), shaken for 1 hour, centrifuged, filtered, and analysed by using a high-performance liquid chromatography system (Gilson) equipped with an ion exchange column (Aminex HPX-87H, Bio-Rad) and a refractive index detector, using 0.03 M sulphuric acid as mobile phase at 42°C. Chromatograms were analysed using Chromax 2007 software, version 1.0a (PoL-lab, Poland). At the same time, standard sugar solutions (sucrose, fructose, and glucose) were prepared. The tests were carried out in duplicate. Sugar content was expressed in g per 100 g fresh weight [21].

The 2,6-dichloroindophenol titrimetric method [22] was used to determine the vitamin C content of pulp, peel, and whole fruit extracts. Fresh fruit pulp/peel/whole fruit was homogenized in a blender and the extract was filtered through a paper filter, then through 0.45 *μ*m porosity cellulose acetate membranes. A 5 mL volume of the clear juice obtained was diluted to 50 mL with a metaphosphoric acid-acetic acid solution, and 7 mL was titrated against a standard indophenol solution. Extractions and titrations were performed in triplicate.

The extraction procedure of tannin and its content in citrus pulp, peel, and whole fruit extracts was estimated by the method described by Okwu and Emenike [23]. The extracts (1 mL) were mixed with Folin-Ciocalteu's reagent (0.5 mL), followed by the addition of saturated Na_2_CO_3_ solution (1 mL) and distilled water (8 mL). The reaction mixture was allowed to stand for 30 minutes at room temperature. The supernatant was obtained by centrifugation, and absorbance was recorded at 725 nm using a UV-visible spectrophotometer (Unicam 5625 UV/Vis Spectrophotometer). Tannin content was calculated as *μ*g tannic acid equivalent obtained from a calibration curve. A stock solution of 1 mg/mL tannic acid was prepared in 80% chilled ethanol. It was then diluted ten times and used as a working standard solution. From this stock, 0.1, 0.2, 0.3, 0.4, and 0.5 mL of samples were taken into separate test tubes. Then, 0.5 mL of Folin-Ciocalteu's reagent and 1 mL of saturated sodium bicarbonate solution were added to each of the test tube. The volume of each of the test tube was diluted to 8 mL with distilled water. The absorbance of each sample was measured against a blank reagent.

The extraction procedure of phenolic compounds from citrus fruits was performed according to a modified method from Bakowska-Barczak and Kolodziejczyk [24]. Citrus lyophilisate (0.1 g) was twice extracted with 1 mL of 80% aqueous methanol containing 0.1% formic acid. The mixture was shaken for 60 min (25°C, 300 rpm). Next, it was sonicated for 20 min and centrifuged for 30 min (20817 × g, 4°C). Supernatants were combined, and the final volume of the extract was diluted to 2 mL with extraction solvent. The mixture was halved and used for the analysis of total phenolic content.

Total phenolic content (TPC) was determined according to Song et al. [25]. A 2.5 mL volume of 1 : 10 diluted Folin-Ciocalteu's reagent was added to 0.5 mL of the diluted extract. After 4 min, 2 mL of saturated sodium carbonate solution (about 75 g/L) was added. After 2 h of incubation at room temperature, the absorbance of the mixture was measured at 760 nm.

Levels of gallic acid, p-coumaric acid, caffeic acid, sinapic acid, chlorogenic acid, and ferulic acid were determined using a Gilson high-performance liquid chromatography (HPLC) apparatus with UV/Vis DAD 170 detection, Waters Symmetry C18 RP column 5 *μ*m4.6 × 250 mm, as described by Häkkinen et al. [26]. The procedure of hydrolysis and separation of phenolic acids has been described in detail by Oczkowski et al. [27]. Phenolic acids were separated using a mixture of 1% acetic acid and 50% acetonitrile, and compounds were detected at 320 nm. The comprehensive identification and assignment of each phenolic compound was carried out by comparing retention times of compounds and UV spectra to authentic standards using HPLC [28].

The ABTS^•+^ assay was carried out according to Re et al. [29]. A stock solution of ABTS^+^ was prepared from 7 mM ABTS^•+^ and 2.45 mM potassium persulphate, left in the dark for 16 h at ambient temperature, and used within 2 days. The ABTS^•+^ working solution was prepared by diluting the stock solution with ethanol to an absorbance of 0.70 ± 0.02 at 734 nm. A 0.02 mL volume of each sample was mixed with 0.13 mL deionized water and 1.5 mL ABTS^•+^ working solution. The absorbance of the mixture was measured at 734 nm after 6 min of incubation at room temperature, and the percentage of inhibition of absorbance at 734 nm was calculated (Unicam 5625 UV/Vis Spectrophotometer).

DPPH^•^ free radical scavenging activity was measured by the method developed by Bocco et al. [11]. A 0.05 mL aliquot of the previously diluted sample was added to 1.95 mL of a 0.06 mmol/L DPPH^•^ solution in methanol and mixed. After 25 min, the decrease in the absorbance was measured by spectrophotometry at 515 nm (Unicam 5625 UV/Vis Spectrophotometer). The exact DPPH^•^ concentration remaining in the solution was calculated from a calibration curve. The control consisted of a methanol solution of Trolox at different concentrations. The antioxidant capacity was expressed as mmoL Trolox equivalents/g of FW sample.

Fresh fruit samples were freeze-dried and ground in a ceramic mortar. To minimize the risk of metal contamination, all glassware and utensils were rinsed with tap water, soaked in an acid bath (5 M HNO_3_) for 24 h, rinsed with demineralized water, and dried under a laminar flow hood before use. A 10 mL volume of concentrated HNO_3_ (Sigma-Aldrich) was poured over weighed portions of each sample (usually 500 ± 1 mg), which were then subjected to wet ashing. Samples were mineralized in a Microwave Digestion System in Teflon vials (DAP 100), with optimal temperature and pressure applied to each sample, and monitored throughout the acid digestion procedure (Berghof Speedwave). Mineralization was performed according to the following scheme: 15 min with the temperature raised from room temperature up to 140°C, 5 min at a stable temperature of 140°C, 5 min with the temperature raised from 140°C up to 170°C, 15 min at 170°C, and finally cooling to room temperature (variable time). The pressure throughout the mineralization process did not exceed 12 bar (1.2 MPa). A clear solution was obtained when the mineralization process was completed. Next, the solution was cooled to room temperature and transferred to a 50 mL volumetric flask filled with demineralized water (ELGA Pure Lab Classic) to the 50 mL mark. Elements were determined with an ICP-OES (Inductively Coupled Plasma Optical Emission Spectrometer; Thermo Scientific iCAP Series 6500) equipped with a charge injection device detector. The spectrometer was controlled by a PC-based iTEVA software. The following instrumental parameters were used: RF generator power = 1150 W; RF generator frequency = 27.12 MHz; coolant gas flow rate = 16 L min^−1^; carrier gas flow rate = 0.65 L min^−1^; auxiliary gas flow rate = 0.4 L min^−1^; max integration time = 15 s; pump rate = 50 rpm; viewing configuration = axial; replicate = 3, and flush time = 20 s. Periodic table mix 1 for ICP, TraceCERT 10 mg/L, Fluka multielement stock solution (Inorganic Ventures) was used as a standard.

### 2.4. Statistical Analysis

Statistical data analysis was carried out using the commercial software Statistica, version 13.3.

Tables 1–5 present the results of two-way analysis of variance (ANOVA). The mathematical model takes into account the influence of the following fixed factors on the content of different parameters in the sample: S—the fruit species (orange, pomelo, mandarin, etc.) and P—the part of the fruit (pulp (F) or peel (P)).

(1) $$\begin{array}{c} \text{Yijk}=\mu +\text{Si}+\text{Pj}+\text{eijk}, \end{array}$$

where *μ* is the grand mean, Si is the fixed effect of the fruit species, Pj is the fixed effect of the part of the fruit, eijk is the residual effect.

Tables 1–5 also present the results of one-way analysis of variance (ANOVA) used to determine the fixed effect of the species of whole fruit—S on the homogenate content of the whole fruit (W) on the different parameter contents in sample a was used.

(2) $$\begin{array}{c} \text{Yijk}=\mu +\text{Si}+\text{eij}, \end{array}$$

where *μ* is the grand mean, Si is the fixed effect of the fruit species, eijk is the residual effect.

Significance of differences between means was determined using the Duncan test, assuming significance levels of *p* = 0.05 and *p* = 0.01.

Table 6 presents Pearson's correlation analysis between the content of ABTS^•+^ or DPPH^•^ and that of phenolic compounds, ascorbic acid, and tannins for the pulp (F), peel (P), and whole (W) citrus fruits.

## 3. Results and Discussion

### 3.1. Dietary Value of Citrus Fruits

Citrus fruits are considered a valuable source of health-promoting substances that effectively coordinate and stimulate metabolic changes [25, 30]. They have a low glycaemic index, owing to which they are used in a number of diets. The glycaemic index is significantly influenced by the presence of sugars (glucose, fructose, and sucrose) and dietary fibre. The content and type of simple sugars depend not only on the variety, but also on the part of the fruit [31].

In the pulp of the citrus fruits, the content of glucose ranged from 0.137 g/100 g in mandarins to an average of 0.270 g/100 g in red grapefruit and lime, and the differences between them were statistically significant (Table 1). The level of fructose in the citrus fruit pulp was markedly higher than that of glucose. Its highest content was noted in the orange pulp (7.01 g/100 g), and the lowest average content (1.08 g/100 g) was noted in the lemon and lime pulp (*p* ≤ 0.05). Lemon pulp (0.048 g/100 g) had the lowest sucrose content, while pomelo pulp had the highest (6.12 g/100 g). Similar results were obtained by Jamil et al. [31], who reported a significantly lower content of sugars in limes than in other citrus fruits.

The peel of the fruits was markedly less rich in sugars (glucose, fructose, and sucrose) than the pulp (F *vs*. P; *p* < 0.001; Table 1). This translated into lower concentrations in the whole fruits than in the pulp (F *vs*. W). This indicates that the dietary value of products can be improved by using whole citrus fruits. Exceptions are orange and mandarin, in which glucose content was significantly higher in the peel (0.304 g/100 g and 0.249 g/100 g, respectively) than in the pulp (0.236 g/100 g and 0.137 g/100). Sucrose content was also higher in the peel *vs*. the pulp of mandarins (0.997 g/100 *vs*. 0.065 g/100) and limes (0.182 g/100 *vs*. 0.130 g/100; Table 1). This caused a slight increase in the content of these sugars in the whole fruit (W) compared to the pulp (F). Therefore, in the case of mandarins and oranges, it may not be nutritionally beneficial to use the whole fruit in the production of processed foods. The research also shows that in the case of whole fruits (W), oranges had significantly the highest level of fructose and glucose (5.52 g/100 g and 0.264 g/100 g, respectively). Pomelos (4.21 g/100 g) had the highest content of sucrose, while whole lemons proved to be the least rich in sugars (Table 1).

Dietary fibre is a valuable nutrient for maintaining good health. It is used in the prevention of numerous diseases, including metabolic syndrome diseases [2, 10]. Dietary fibre content in the citrus fruit pulp ranged from 26.32 g/100 g (mandarin) to 33.0 g/100 g (average for the grapefruit cultivars and pomelo); the differences were statistically significant. In the peel of the citrus fruits, the fibre content was about twice as high as in the pulp, ranging from 60.05 g/100 g in white grapefruit to 64.10 g/100 g (average for lemon, lime, and orange); the differences were statistically significant. This resulted in significantly higher fibre content in the whole fruits than in the pulp (F *vs*. W; *p* < 0.001; Table 1). This increase reached over 40% in the case of lemon and lime. This may encourage the use of whole fruit for processing of products such as jams, mousses, or nectars and thus contribute to an increase in fibre intake. Furthermore, citrus fibre exhibits bioactive functions due to the presence of compounds similar in structure to polyphenols, and thus may be used as an effective antioxidant to extend the shelf life of products [32].

### 3.2. Antioxidant Properties of the Samples

Citrus fruits have a high content of ascorbic acid. The content of this vitamin is considered a basic indicator of the quality and nutritional value of fruits and fruit products [30]. Although ascorbic acid has lower antioxidant activity than phenolic compounds, including phenolic acids, tannins, catechins, and anthocyanins, its multifaceted antioxidant activity should be stressed [2, 33].

According to Martí et al. [34], the content of ascorbic acid in citrus fruit depends on the species and part of the fruit, which was confirmed in the present study (Table 2). In the pulp of the fruits, the highest content of ascorbic acid was noted in pomelo (72.26 mg/100 g) and the lowest was noted in the grapefruit cultivars, particularly red and white (22.42 and 17.19 mg/100 g, respectively) (Table 2). Thus, it is worth noting that just 100 g of pomelo pulp meets the adult daily requirement for this vitamin [35]. The results were consistent with the research of Dhuique-Mayer et al. [36]. However, due to the many agrotechnical and climatic variables associated with the cultivation of citrus fruits, as well as variation in the degree of ripeness, it is unsurprising that most researchers report a wide range of ascorbic acid levels in various citrus fruits [6].

In the peel of citrus fruits, the highest content of ascorbic acid was found in green grapefruit (67.36 mg/100 g) and the lowest was found in lemon (7.83 mg/100 g), and the differences between them were statistically significant (*p* < 0.05; Table 2). It is worth noting that the lemon pulp contained more than 7 times as much ascorbic acid as the peel (*p* < 0.001). Lower ascorbic acid content in peel *vs*. pulp was also found in oranges, pomelos, mandarins, and limes, while the reverse relationship was found in all grapefruit varieties (*p* < 0.001; Table 2). Whole red grapefruits had the lowest ascorbic acid content (27.17 mg/100 g) of all the citrus fruits, while the highest concentration of ascorbic acid was recorded in the whole pomelo fruit (47.45 mg/100 g). This was probably due to the content of this vitamin in the pulp, which was more than 4.5 times as high as in the peel (F *vs*. P; *p* < 0.001; Table 2). Comparison of the content of ascorbic acid in whole fruit *vs*. pulp shows that the use of whole grapefruits of all cultivars in the food industry increases their nutritional value in terms of ascorbic acid content. The differences in the content of ascorbic acid in the whole grapefruits and their pulp ranged from over 20% for red grapefruit (*p* = 0.024) to nearly 90% for white grapefruit (*p* < 0.001).

Citrus fruits also owe their antioxidant properties to the presence of tannins. In addition to their antioxidant capacity (free radical scavenging and metal chelation), tannins are also believed to have health-beneficial effects in the treatment and prevention of cancer, cardiovascular diseases, and other pathologies [5, 6, 37]. However, as tannins impart a bitter and astringent taste, they may reduce the appeal of citrus fruits for consumers. According to Okwu and Emenike [23], the level of tannins in citrus fruit ranges from 0.01 to 0.04 mg/100 g. Among the fruits tested, the highest content of tannins was found in green grapefruit pulp (26.09 *μ*g/100 g), and the lowest was found in pomelo pulp (4.49 *μ*g/100 g). The peel of all citrus fruits was significantly richer in tannins than the pulp. The highest level was noted in white grapefruit peel (40.95 *μ*g/100 g), and the lowest was noted in orange peel (22.80 *μ*g/100 g). The greatest (sevenfold) difference in tannin content was found between the pulp and peel of pomelos (F *vs*. P; *p* < 0.001) (Table 2).

The significantly higher concentration of tannins in the citrus peel corresponded to their increased content in the whole fruit compared to the pulp (F *vs*. W). The increase in tannin content in whole fruits compared to the pulp is particularly visible in the case of red and white grapefruit, reaching 82% and 99%, respectively (F *vs*. W; *p* < 0.001). The lowest level of these compounds was found in whole oranges and pomelos (on average 16.18 *μ*g/100 g; Table 2). Due to the bitter aftertaste associated with the higher content of tannins in the peel, this may limit the use of whole grapefruit, orange, or pomelo, but it increases the health-promoting properties of the product [37].

Phenolic compounds are classified as chemopreventive substances that enhance the body's natural defence mechanisms against oxidative stress. Owing to their antioxidant properties, they neutralize chemical oxidants, free radicals, and environmental carcinogens, thus preventing damage to genetic material [2, 5].

The total content of phenolic compounds in the pulp of the citrus fruits ranged from an average of 62 mg GAE/100 g in pomelo and green grapefruit to an average of 167 mg GAE/100 g in orange and red grapefruit; the difference between them was statistically significant. The content of phenolic compounds in the peel of all citrus fruits was significantly higher than in the pulp (F *vs*. P; *p* < 0.001). The greatest (tenfold) difference between the peel and the pulp was noted in pomelos, and the lowest (about 23%) was noted in red grapefruit (Table 2). At the same time, the highest level of phenolic compounds was noted in the peel of pomelo and white grapefruit (average 648 mg GAE/100 g), and the lowest was noted in the peel of mandarin and red grapefruit (average 206.4 mg GAE/100 g). The results are consistent with the research of Ramful et al. [38], who analysed 21 varieties of citrus fruit and obtained phenolic compound content in the peel ranging from 188.2 ± 6.5 to 776.7 ± 5.7 mg GAE/100 g FW. Li et al. [39] reported that grapefruit peels have higher total phenolic content than mandarin, Yen Ben lemon, Meyer lemon, and orange peel. In addition, the high content of phenolic compounds in the peels has been shown to protect the fruits and vegetable against UV light, pathogens, and predators. According to Barros et al. [7], peels contain higher concentrations of these compounds because they are the outer part of the fruit, and thus are more predisposed to the synthesis of phenolic compounds. Many studies indicate higher levels of phenolic compounds in citrus peel compared to the pulp [1, 7, 40].

Therefore, citrus peels as a source of valuable phenolic compounds can be used in food products as active ingredients or even as replacements for synthetic preservatives, such as butylated hydroxyanisole and butylated hydroxytoluene [41], which improves the health-promoting value of the products.

Analysis of the content of phenolic compounds in whole fruits (W) compared to their pulp (F) showed that it was significantly higher in the whole fruits in all cases. This was due to their higher concentration in the peel (F *vs*. P; *p* < 0.001; Table 2). Such relationships were noted by Elkhatim et al. [3] for grapefruit and orange.

Antioxidant activity is largely dependent on the presence of phenolic acids. Phenolic acids have been shown to have the highest antioxidant potential of all polyphenols. Citrus fruits, however, are not a rich source of phenolic acids. For this reason, in the present study, phenolic acids whose content is relatively high and which have high antioxidant potential were selected for analysis, based on literature data [42, 43].

Chlorogenic acid was the predominant phenolic acid in all fruits, in both the pulp and the peel, while the content of gallic acid was the lowest. However, depending on the cultivar and part of the fruit, the content of individual acids and their proportions varied (Table 3).

The red grapefruit pulp contained significantly the highest amount of chlorogenic acid (74.91 mg/100 g), caffeic acid (14.15 mg/100 g), and ferulic acid (29.16 mg/100 g). The content of chlorogenic acid was also significantly higher in the mandarin pulp than in the other citrus fruits (72.02 mg/100 g; Table 3). Chlorogenic acid (the ester of caffeic and quinic acid) exhibits relatively high antioxidant activity [41], which is linked to its chemical structure, specifically to the number of hydroxyl groups and the degree of their esterification. In addition to citrus fruit, it has also been found in high amounts in coffee and in blueberries, apples, pears, and eggplant [7].

In addition to chlorogenic acid, other phenolic acids present in significant quantities in the pulp of the citrus fruits were ferulic, p-coumaric, and caffeic acids. In lemon pulp, the predominant acids were sinapic acid (32.74 mg/100 g) and p-coumaric acid (30.93 mg/100 g). The high content of these acids is particularly important due to their high antioxidant potential. The results were consistent with the research of Xi et al. [1]. According to Bocco et al. [11], the most abundant phenolic acids in oranges were ferulic > sinapic > p-coumaric > caffeic acid; the authors did not assay the content of chlorogenic acid.

The differences in the content of individual phenolic acids between the peel and the pulp varied depending on the cultivar of citrus fruits (Table 3). A significantly higher content of gallic acid in the peel *vs*. the pulp was noted in the oranges and mandarins; chlorogenic acid in lemon, lime, and white grapefruit; and caffeic acid in orange, mandarin, lemon, and green grapefruit (*p* < 0.001). The content of coumaric acid and ferulic acid was significantly higher in the peel *vs*. the pulp of all fruits tested (except for coumaric acid in lemon). Sinapic acid was found only in lemon pulp and in both parts (peel and pulp) of orange and red grapefruit. Its content was significantly higher in the peel of orange and red grapefruit than in the pulp (*p* < 0.001; Table 3).

As in the case of the peel and pulp, chlorogenic acid had the highest share among phenolic acids in the whole fruits (W). The highest content per 100 g of fruit was noted in red and white grapefruit, with 62.57 mg and 64.85 mg, respectively. Whole oranges and grapefruits had the highest content of sinapic acid among all fruits (34.06 mg/100 g on average), and whole oranges had the highest content of caffeic acid (22.74 mg/100 g). These levels were higher than in the pulp (F *vs*. W; *p* < 0.001; Table 3). Whole limes had the most coumaric acid (27.29 mg/100 g) of all fruits and, as in the case of chlorogenic acid, its quantity was statistically significantly higher in the whole fruit than in the pulp (F *vs*. W; *p* < 0.001). The concentration of ferulic acid in whole red grapefruits (W) was significantly higher than in the other citrus fruits, and also almost twice as high as in the pulp of this fruit (F *vs*. W; *p* < 0.001; Table 3).

### 3.3. Antioxidant Activity

The antioxidant properties of citrus fruits are manifested by their ability to reduce the amount of free radicals formed during oxidation processes. To determine the antioxidant properties of citrus pulp, peel, and whole fruit, methods involving two types of radicals were used: DPPH^•^ and ABTS^•+^ (Table 4). Analysis of the antioxidant capacity of the pulp of citrus fruits using ABTS^•+^ radicals showed that it was by far the highest in the orange pulp (247.4 mmol Trolox/g), and the difference compared to the pulp of the other fruits was statistically significant (*p* < 0.05). The antioxidant activity of orange pulp was also significantly higher than that of the peel (F *vs*. P; *p* < 0.001). The same relationship was also noted for pomelos and all grapefruit varieties. In the case of mandarin, lemon, and lime, the antioxidant capacity determined using the ABTS^•+^ radical was significantly higher in the peel than in the pulp (F *vs*. P; *p* < 0.001).

The higher antioxidant capacity of the mandarin and lemon peels determined using ABTS^•+^ radicals translated into a higher value in the whole fruit relative to the pulp (F *vs*. W; *p* < 0.001). The highest antioxidant capacity against the ABTS^•+^ radical was determined for whole oranges and mandarins (on average 182.7 mmol Trolox/g), and it was significantly higher than for the other fruits (*p* < 0.05; Table 4).

The highest DPPH^•^ radical scavenging ability was found in pomelo pulp (297.1 mmol Trolox/g), and the lowest was found in lemon pulp (12.25 mmol Trolox/g). For pomelos, oranges, and all grapefruit varieties, the ability to scavenge the DPPH^•^ radical was significantly higher in the pulp than in the peel (F *vs*. P; *p* < 0.001). The reverse relationship was found for mandarin, lemon, and lime (F *vs*. P; *p* < 0.001).

Whole mandarins, lemons, and limes had a higher DPPH^•^ radical scavenging capacity than their pulp (F *vs*. W; *p* < 0.001). It is worth noting that whole lemons (223.7 mmol Trolox/g), mandarins (213.4 mmol Trolox/g), and pomelos (219.6 mmol Trolox/g) had significantly the highest DPPH^•^ radical scavenging capacity of all analysed fruits (*p* ≤ 0.05; Table 4). The results were consistent with the research of Barros et al. [7].

### 3.4. Heavy Metal Content

Many reports indicate that the use of whole fruit, including the peel, entails the risk of intake of harmful substances potentially present in the peel, such as heavy metals [13]. The level of lead in the citrus fruits analysed (pulp, peel, and whole fruit) ranged from 0.354 *μ*g/100 g in mandarin pulp to 3.01 *μ*g/100 g in red grapefruit peel. Cadmium content was much lower, ranging from 0.004 *μ*g/100 g in lime pulp to 0.199 *μ*g/100 g in green grapefruit peel (Table 5). The values did not exceed the acceptable levels according to WHO [43], i.e., 10-20 *μ*g/100 g fresh weight of fruit for lead and 0.050 mg/kg for cadmium [43]. Results of this study are not compatible with those of Mausi et al. [44] who studied the concentration of heavy metals (lead and cadmium) in fresh orange from Eldoret in Kenya and reported concentrations above the standard level. Also, the results of Kalagbor and Diri [45] from a study on a farm in Kaani, Bori, and the Rivers State in Nigeria, showed that the concentration of lead in orange is above standard level. The discrepancies between the results of their studies and the results of this study could be attributed to different culture conditions and different soil compositions.

The content of heavy metals depended on the species (or cultivar) and part of the fruit (Table 5). The highest concentration of lead (2.47 *μ*g/100 g) was noted in red grapefruit pulp, and the highest level of cadmium (0.116 *μ*g/100 g) was noted in white grapefruit pulp. The lowest level of lead was found in mandarin pulp (0.354 *μ*g/100 g), while cadmium content was lowest in lime pulp (0.004 *μ*g/100 g). The highest content of lead was found in pomelo and red grapefruit (and in its pulp), and the lowest was found in orange peel (1.02 *μ*g/100 g). The content of cadmium was significantly the highest in the peel of red and green grapefruit (average 0.194 *μ*g/100 g) and the lowest in the peel of orange, mandarin, lemon, and lime (average 0.051 *μ*g/100 g; Table 5).

The content of lead and cadmium in the citrus peels was significantly higher than that in the pulp (F *vs.* W; Table 5). This is in line with other research [13]. According to Saleh et al. [13], fruit peels accumulate higher quantities of heavy metals than the pulp. For this reason, it is often recommended to eat only the pulp of fruit. The results of the present study show that while the content of the metals tested is in fact higher in whole fruit than in the pulp, the levels are far below the recommended WHO [46] guidelines, which indicate that the use of whole fruit in production is safe for consumers.

### 3.5. Correlation Coefficients

The antioxidant capacity of fruits is attributed to the presence of phenolic compounds, ascorbic acid [42, 47], and tannins. Because it is difficult to assess to what extent individual groups of compounds affected the degree of inhibition of ABTS^•+^ or DPPH^•^ radicals, a direct correlation coefficient was determined between the capacity to inhibit ABTS^•+^ and DPPH^•^ radicals and the content of phenolic compounds, ascorbic acid, and tannins using Pearson's coefficient at *p* < 0.01 and *p* < 0.05 (Table 6). In the pulp, a significantly positive coefficient of correlation was found between the ability to inhibit DPPH^•^ radical and phenolic compounds (in mandarin pulp), tannins (in red grapefruit pulp), and ascorbic acid (in pomelo pulp). Literature data indicate that tannins are probably the compounds most responsible for the suppressive effect on stable DPPH^•^ radicals [48], which was not found in the present study. Phenolic compounds also did not have a clear inhibitory effect on DPPH^•^ or ABTS^•+^ radicals. Perhaps this is due to the varied content of phenolic acids in individual citrus fruits.

Many more dependencies were found in the peel of the fruits. A significantly positive coefficient of correlation in relation to ABTS^•+^ radical inhibition ability was found for phenolic compounds and ascorbic acid (in pomelo peel) and in relation to DPPH^•^ scavenging ability for tannins (in lime peel) and ascorbic acid (in orange, mandarin, and lime peel). In whole pomelos, there was a significantly positive coefficient of correlation between ABTS^•+^ radical inhibition ability and the presence of ascorbic acid, and in whole oranges, an analogical coefficient of correlation was observed between DPPH^•^ radical inhibition capacity and ascorbic acid (Table 6). Similar research was conducted by Ghasemia et al. [14] and Pretel and Botella [47]. The positive correlation between the ability to inhibit radicals and the content of ascorbic acid indicated that it may be one of the main factors contributing to the antioxidant capacity of these fruits. Most studies, however, indicate that phenolic compounds have a greater effect on antioxidant processes in plant sources or a combination of phytochemicals [33, 49]. The conflicting results could be influenced by a number of factors, such as the variety of the fruit, its ripeness, or the analytical methods used in different studies to assess antioxidant power [50].

## 4. Conclusions

The study carried out on eight cultivars of citrus fruit showed significant differences in the distribution of bioactive substances, as well as heavy metals and antioxidant potential, between the peel and the pulp. The peels of pomelo, lemon, and all grapefruit cultivars had markedly less glucose, fructose, and sucrose contents than the pulp. The contents of fibre, tannins, and phenolic compounds in all citrus fruits were significantly higher in the peel than in the pulp, especially in the case of pomelo. Thus, the use of whole citrus fruits, and not just the pulp, may increase health benefits, due to the far lower sugar content and higher content of dietary fibre and phenolic compounds, including ferulic acid, in the whole fruit than in the pulp. This is particularly beneficial in the case of whole grapefruits, which additionally had a higher concentration of ascorbic acid. Whole lemons, limes, and especially mandarins also had higher antioxidant potentials than their pulps, due to their higher content of ascorbic acid, tannins, and phenolic compounds.

It was not confirmed that the use of whole fruit, including the peel, poses a health risk resulting from increased uptake of cadmium and lead. The levels of these metals in the whole fruit, while higher than in the pulp, were significantly below the acceptable daily intake.

Therefore, in terms of prevention of numerous diseases of civilization, it seems that whole citrus fruits should be used in the processing of citrus fruit products.

## Figures and Tables

**Table 1:** Content of dietary fibre and sugar (g/100 g) in citrus pulp (F), peel (P), and whole fruits (W).

Fruit/index	Part	Orange	Pomelo	Mandarin	Lemon	Key lime	Red grapefruit	Green grapefruit	White grapefruit
Glucose	F	0.236 ± 0.004^b^	0.205 ± 0.005^c^	0.137 ± 0.012^d^	0.244 ± 0.009^ab^	0.272 ± 0.008^a^	0.269 ± 0.007^a^	0.207 ± 0.005^c^	0.215 ± 0.011^c^
	P	0.304 ± 0.005^a^	0.111 ± 0.011^d^	0.249 ± 0.012^b^	0.160 ± 0.003^c^	0.185 ± 0.004^c^	0.099 ± 0.005^d^	0.180 ± 0.003^c^	0.160 ± 0.003^c^
	W	0.264 ± 0.031^a^	0.161 ± 0.048^b^	0.193 ± 0.062^b^	0.204 ± 0.047^b^	0.232 ± 0.051^ab^	0.194 ± 0.091^b^	0.193 ± 0.013^b^	0.180 ± 0.018^b^

Fructose	F	7.01 ± 0.376^a^	2.35 ± 0.068^c^	2.34 ± 0.048^c^	1.02 ± 0.078^d^	1.14 ± 0.041^d^	2.66 ± 0.091^c^	3.17 ± 0.089^b^	2.25 ± 0.048^c^
	P	3.45 ± 0.160^a^	0.692 ± 0.019^e^	1.40 ± 0.115^b^	0.444 ± 0.029^f^	0.476 ± 0.013^f^	0.673 ± 0.024^e^	0.854 ± 0.033^d^	0.990 ± 0.045^c^
	W	5.52 ± 0.801^a^	1.52 ± 0.060^b^	1.93 ± 0.009^b^	0.748 ± 0.092^c^	0.820 ± 0.096^c^	1.77 ± 0.045^b^	2.01 ± 0.018^b^	1.62 ± 0.020^b^

Sucrose	F	2.24 ± 0.087^d^	6.12 ± 0.078^a^	0.065 ± 0.005^f^	0.048 ± 0.003^g^	0.130 ± 0.006^e^	5.22 ± 0.156^b^	5.12 ± 0.071^b^	2.77 ± 0.063^c^
	P	0.569 ± 0.043^c^	1.86 ± 0.059^a^	0.997 ± 0.098^b^	0.041 ± 0.003^f^	0.182 ± 0.007^de^	0.235 ± 0.012^d^	0.192 ± 0.005^de^	0.170 ± 0.008^e^
	W	1.52 ± 0.060^c^	4.21 ± 0.433^a^	0.481 ± 0.064^d^	0.052 ± 0.009^e^	0.013 ± 0.006^f^	2.95 ± 0.079^b^	2.97 ± 0.056^b^	1.35 ± 0.052^c^

Dietary fibre	F	28.74 ± 1.07^b^	33.11 ± 1.32^a^	26.32 ± 0.643^c^	28.29 ± 0.454^b^	28.85 ± 0.786^b^	32.96 ± 0.762^a^	33.65 ± 0.609^a^	33.61 ± 0.746^a^
	P	63.77 ± 0.591^a^	61.85 ± 0.957^bc^	62.53 ± 0.477^b^	64.64 ± 0.591^a^	63.89 ± 0.394^a^	61.22 ± 0.926^c^	61.07 ± 0.621^c^	60.05 ± 0.975^d^
	W	48.27 ± 1.48^c^	52.62 ± 0.935^b^	37.99 ± 1.55^d^	37.88 ± 0.653^d^	34.55 ± 1.26^e^	55.05 ± 1.47^a^	48.81 ± 0.970^c^	51.88 ± 0.633^b^

Statistical analysis	*p*_value_								

F^1^*vs.* P									

Glucose		<0.001	<0.001	<0.001	<0.001	<0.001	0.048	<0.001	<0.001
Fructose		<0.001	<0.001	<0.001	<0.001	<0.001	<0.001	<0.001	<0.001
Sucrose		<0.001	<0.001	<0.001	<0.001	<0.001	<0.001	<0.001	<0.001
Dietary fibre		<0.001	<0.001	<0.001	<0.001	<0.001	<0.001	<0.001	<0.001

F^2^*vs.* W									

Glucose		0.146	0.022	0.059	0.033	0.041	0.034	0.014	<0.001
Fructose		0.002	<0.001	0.002	<0.001	<0.001	<0.001	<0.001	<0.001
Sucrose		<0.001	<0.001	0.022	0.872	0.326	0.003	0.001	<0.001
Dietary fibre		<0.001	<0.001	<0.001	<0.001	<0.001	<0.001	<0.001	<0.001

^a,b,c,d,e,f^Values in rows with different letters are significantly different at *p* ≤ 0.05: F rows—superscript letters indicate statistically significant differences in fibre and sugar content in the pulp (F) between citrus cultivars; P rows—superscript letters indicate statistically significant differences in fibre and sugar content in the peel (P) between citrus cultivars. W rows—superscript letters indicate statistically significant differences in fibre and sugar content in the whole fruit (W) between citrus cultivars. ^1^F *vs*.P‐*p*_value_—level of significance of differences between pulp (F) and peel (P) of individual citrus cultivars. ^2^F *vs*.W‐*p*_value_—level of significance of differences between pulp (F) and whole fruit (W) of individual citrus cultivars.

**Table 2:** Content of phytochemicals in citrus pulp (F), peel (P), and whole fruits (W).

Fruit/index	Part	Orange	Pomelo	Mandarin	Lemon	Key lime	Red grapefruit	Green grapefruit	White grapefruit
Phenolic compounds (mg GAE/100 g)	F	164.37 ± 13.35^a^	63.07 ± 7.88^e^	99.53 ± 9.31^c^	78.59 ± 7.46^d^	105.1 ± 6.98^c^	169.6 ± 7.86^a^	61.87 ± 7.28^e^	135.2 ± 6.14^b^
	P	312.2 ± 20.33^b^	645.0 ± 21.48^a^	204.3 ± 15.43^e^	251.1 ± 15.80^d^	282.7 ± 11.17^c^	208.7 ± 13.81^e^	238.7 ± 7.08^de^	651.3 ± 17.18^a^
	W	240.68 ± 15.66^c^	466.5 ± 14.10^a^	158.7 ± 14.22^ef^	174.4 ± 17.37^e^	196.3 ± 16.87^d^	238.3 ± 34.64^cd^	139.1 ± 14.06^f^	416.0 ± 17.00^a^

Ascorbic acid (mg/100 g)	F	50.71 ± 5.21^c^	72.26 ± 6.11^a^	42.04 ± 2.90^d^	59.42 ± 3.87^b^	41.13 ± 3.05^d^	22.42 ± 3.34^f^	28.92 ± 3.31^e^	17.19 ± 1.59^f^
	P	30.33 ± 3.52^c^	16.09 ± 1.88^e^	31.64 ± 2.68^c^	7.83 ± 0.398^f^	24.35 ± 2.18^d^	33.50 ± 2.94^c^	67.36 ± 2.49^a^	43.79 ± 3.14^b^
	W	42.50 ± 3.66^ab^	47.45 ± 3.38^a^	39.02 ± 3.08^bc^	36.58 ± 5.84^bc^	33.22 ± 2.25^c^	27.17 ± 2.36^d^	43.32 ± 4.39^ab^	32.10 ± 2.44^c^

Tannins (*μ*g/100 g)	F	10.13 ± 0.479^d^	4.49 ± 0.190^f^	9.74 ± 0.793^e^	8.59 ± 0.857^e^	13.54 ± 0.457^c^	14.11 ± 0.477^b^	26.09 ± 0.581^a^	14.13 ± 0.445^b^
	P	22.80 ± 1.78^f^	31.5 ± 1.58^c^	29.9 ± 1.75^cd^	28.04 ± 1.39^de^	34.81 ± 4.22^b^	34.94 ± 0.757^b^	27.38 ± 0.778^e^	40.95 ± 1.43^a^
	W	16.21 ± 1.26^d^	16.15 ± 1.72^d^	20.38 ± 0.925^c^	19.42 ± 1.52^c^	22.83 ± 1.54^b^	25.75 ± 1.73^a^	26.26 ± 1.54^a^	28.25 ± 1.52^a^

Statistical analysis	*p*_value_								

F^1^*vs.* P									

Phenolic compounds		<0.001	<0.001	<0.001	<0.001	<0.001	<0.001	<0.001	<0.001
Ascorbic acid		<0.001	<0.001	<0.001	<0.001	<0.001	<0.001	<0.001	<0.001
Tannins		<0.001	<0.001	<0.001	<0.001	<0.001	<0.001	<0.001	<0.001

F^2^*vs.* W									

Phenolic compounds		0.050	0.003	<0.001	<0.001	<0.001	0.037	<0.001	<0.001
Ascorbic acid		0.039	<0.001	0.050	<0.001	<0.001	0.024	<0.001	<0.001
Tannins		<0.001	<0.001	<0.001	<0.001	<0.001	<0.001	0.772	<0.001

^a,b,c,d,e,f^Values in rows with different letters are significantly different at *p* ≤ 0.05: F rows—superscript letters indicate statistically significant differences in phytochemical composition in the pulp (F) between citrus cultivars; P rows—superscript letters indicate statistically significant differences in phytochemical composition in the peel (P) between citrus cultivars. W rows—superscript letters indicate statistically significant differences in phytochemical composition in the whole fruit (W) between citrus cultivars. ^1,2^Legend: see Table 1.

**Table 3:** Content of phenolic acids (mg/100 g) in citrus pulp (F), peel (P), and whole fruits (W).

Fruit/index	Part	Orange	Pomelo	Mandarin	Lemon	Key lime	Red grapefruit	Green grapefruit	White grapefruit
Gallic acid	F	4.02 ± 0.905^c^	3.74 ± 0.132^c^	7.65 ± 0.209^b^	9.27 ± 0.185^a^	2.85 ± 0.107^e^	3.33 ± 0.134^d^	3.85 ± 0.186^c^	3.29 ± 0.222^d^
	P	6.54 ± 0.565^c^	2.66 ± 0.157^de^	8.48 ± 0.246^a^	7.77 ± 0.238^b^	2.85 ± 0.189^d^	2.55 ± 0.128^de^	2.50 ± 0.179^de^	2.31 ± 0.080^e^
	W	5.26 ± 1.37^b^	3.31 ± 0.538^c^	7.94 ± 0.410^a^	8.68 ± 0.701^a^	2.77 ± 0.112^c^	3.01 ± 0.393^c^	3.22 ± 0.637^c^	2.66 ± 0.494^c^

Chlorogenic acid	F	56.61 ± 5.73^b^	45.86 ± 5.44^c^	72.02 ± 5.04^a^	8.07 ± 0.409^e^	30.54 ± 5.05^d^	74.91 ± 6.65^a^	42.02 ± 7.46^c^	51.90 ± 7.38^b^
	P	26.07 ± 3.66^d^	24.16 ± 3.91^d^	45.12 ± 5.02^c^	20.86 ± 4.61^d^	64.65 ± 3.57^b^	52.40 ± 4.91^bc^	23.50 ± 4.91^d^	75.68 ± 6.60^a^
	W	41.56 ± 6.23^c^	37.31 ± 3.32^c^	52.77 ± 7.77^b^	15.50 ± 2.30^d^	46.88 ± 7.29^bc^	62.57 ± 8.72^a^	36.97 ± 5.64^c^	64.85 ± 9.74^a^

Caffeic acid	F	9.70 ± 0.878^b^	10.27 ± 0.659^b^	0.248 ± 0.281^e^	9.87 ± 0.419^b^	3.02 ± 0.283^d^	14.15 ± 0.932^a^	6.04 ± 0.162^c^	6.85 ± 0.391^c^
	P	40.86 ± 2.82^a^	1.02 ± 0.062^e^	10.67 ± 1.83^c^	23.03 ± 2.89^b^	2.05 ± 0.066^e^	6.85 ± 0.281^d^	12.44 ± 1.73^c^	2.90 ± 0.119^e^
	W	22.74 ± 1.84^a^	5.78 ± 0.348^e^	6.09 ± 1.14^e^	16.82 ± 1.75^b^	2.54 ± 0.450^f^	11.14 ± 1.31^c^	9.17 ± 0.712^d^	4.92 ± 0.480^e^

p-Coumaric acid	F	11.38 ± 0.835^c^	5.01 ± 0.398^e^	2.05 ± 0.105^f^	30.93 ± 1.94^a^	15.26 ± 0.725^b^	7.80 ± 0.261^d^	5.10 ± 0.109^e^	8.44 ± 0.330^d^
	P	12.56 ± 2.05^c^	29.51 ± 2.24^b^	9.10 ± 0.550^d^	9.56 ± 0.419^d^	36.84 ± 2.12^a^	9.10 ± 0.439^d^	10.53 ± 1.74^cd^	9.09 ± 0.420^d^
	W	12.07^d^	17.75^c^	5.40^f^	20.20^b^	27.29^a^	8.36^e^	7.59^e^	8.88^e^
		±1.54	±0.830	±0.328	±1.02	±1.27	±0.619	±1.01	±0.726

Ferulic acid	F	10.64 ± 1.34^c^	6.17 ± 0.466^d^	12.85 ± 1.70^c^	19.61 ± 1.03^b^	21.03 ± 2.98^b^	29.16 ± 4.07^a^	7.66 ± 1.58^d^	3.53 ± 0.287^e^
	P	38.11 ± 5.32^b^	12.83 ± 1.17^d^	14.30 ± 1.72^d^	32.59 ± 4.28^b^	25.72 ± 1.61^c^	54.62 ± 7.84^a^	12.63 ± 2.13^d^	18.34 ± 1.62^d^
	W	24.92 ± 3.88^bc^	9.70 ± 0.818^e^	14.71 ± 1.02^d^	28.13 ± 2.00^b^	22.16 ± 2.22^c^	56.70 ± 5.53^a^	11.31 ± 1.28^de^	10.23 ± 0.882^e^

Sinapic acid	F	27.87 ± 2.52^b^	0.00	0.00	32.74 ± 2.12^a^	0.00	23.39 ± 2.73^b^	0.00	0.00
	P	41.39 ± 2.26^a^	0.00	0.00	0.00	0.00	30.43 ± 2.61^b^	0.00	0.00
	W	34.88 ± 2.79^a^	0.00	0.00	14.89 ± 1.58^b^	0.00	33.24 ± 2.40^a^	0.00	0.00

Statistical analysis	*p*_value_								

F^1^*vs.* P									

Gallic acid		<0.001	0.614	<0.001	<0.001	0.988	<0.001	<0.001	<0.001
Chlorogenic acid		<0.001	<0.001	<0.001	<0.001	<0.001	<0.001	<0.001	<0.001
Caffeic acid		<0.001	<0.001	<0.001	<0.001	<0.001	<0.001	<0.001	<0.001
p-Coumaric acid		0.014	<0.001	<0.001	<0.001	<0.001	<0.001	<0.001	<0.001
Ferulic acid		<0.001	<0.001	0.005	<0.001	<0.001	<0.001	<0.001	<0.001
Sinapic acid		<0.001	—	—	<0.001	—	<0.001	—	—

F^2^*vs.* W									

Gallic acid		0.063	0.040	0.085	0.036	0.138	0.046	0.016	0.005
Chlorogenic acid		<0.001	0.002	0.012	<0.001	<0.001	0.045	0.139	0.009
Caffeic acid		<0.001	<0.001	<0.001	<0.001	0.019	<0.001	<0.001	<0.001
p-Coumaric acid		0.989	<0.001	<0.001	<0.001	<0.001	0.032	<0.001	0.138
Ferulic acid		0.008	<0.001	0.023	<0.001	0.439	<0.001	<0.001	<0.001
Sinapic acid		0.083	—	—	<0.001	—	0.001	—	—

^a,b,c,d,e,f^Values in rows with different letters are significantly different at *p* ≤ 0.05: F rows—superscript letters indicate statistically significant differences in phenolic acid content in the pulp (F) between citrus cultivars; P rows—superscript letters indicate statistically significant differences in phenolic acid content in the peel (P) between citrus cultivars. The results of two-way analysis of variance (ANOVA). W rows—superscript letters indicate statistically significant differences in phenolic acid content in the whole fruit (W) between citrus cultivars. ^1,2^Legend: see Table 1.

**Table 4:** Antioxidant capacity determined using ABTS^•+^ and DPPH^•^ (mmol Trolox/g) of citrus pulp (F), peel (P), and whole fruits (W).

Fruit/index	Part	Orange	Pomelo	Mandarin	Lemon	Key lime	Red grapefruit	Green grapefruit	White grapefruit
ABTS^•+^	F	247.4 ± 9.92^a^	117.6 ± 10.48^d^	140.6 ± 7.97^c^	97.96 ± 2.79^e^	99.21 ± 9.89^e^	175.7 ± 8.72^b^	146.0 ± 7.17^c^	156.0 ± 9.62^c^
	P	144.3 ± 7.43^c^	95.34 ± 11.61^d^	218.1 ± 8.14^a^	165.3 ± 8.46^b^	125.9 ± 7.72^c^	76.24 ± 8.93^e^	84.57 ± 8.18^de^	96.46 ± 10.08^d^
	W	193.1 ± 19.40^a^	103.7 ± 16.10^d^	172.3 ± 12.56^a^	125.4 ± 8.72^bc^	109.3 ± 14.02^cd^	135.4 ± 9.77^b^	122.5 ± 12.33^bc^	125.6 ± 9.97^bc^

DPPH^•^	F	99.14 ± 9.25^c^	297.1 ± 10.25^a^	16.16 ± 0.652^d^	12.25 ± 1.35^e^	18.99 ± 3.08^d^	170.0 ± 8.11^b^	106.0 ± 9.63^c^	148.1 ± 6.64^b^
	P	73.35 ± 4.55^d^	121.4 ± 8.15^c^	448.9 ± 9.07^a^	444.8 ± 12.84^a^	173.1 ± 9.15^b^	48.96 ± 5.52^f^	58.88 ± 9.94^ef^	62.12 ± 4.86^de^
	W	91.89 ± 5.64^b^	219.6 ± 13.15^a^	213.4 ± 23.32^a^	223.7 ± 15.69^a^	96.99 ± 8.06^b^	109.7 ± 9.24^b^	99.60 ± 19.24^b^	105.7 ± 16.27^b^

Statistical analysis	*p*_value_								

F^1^*vs.* P									

ABTS^•+^		<0.001	<0.001	<0.001	<0.001	<0.001	<0.001	<0.001	<0.001
DPPH^•^		<0.001	<0.001	<0.001	<0.001	<0.001	<0.001	<0.001	<0.001

F^2^*vs.* W									

ABTS^•+^		0.001	0.059	<0.001	<0.001	0.080	<0.001	<0.001	<0.001
DPPH^•^		0.363	<0.001	<0.001	<0.001	<0.001	<0.001	0.421	<0.001

^a,b,c,d,e,f^Values in rows with different letters are significantly different at *p* ≤ 0.05: F rows—superscript letters indicate statistically significant differences in antioxidant capacity determined by ABTS^•+^ and DPPH^•^ in the pulp (F) between citrus cultivars; P rows—superscript letters indicate statistically significant differences in antioxidant capacity determined by ABTS^•+^ and DPPH^•^ in the peel (P) between citrus cultivars; W rows—superscript letters indicate statistically significant differences in antioxidant capacity determined by ABTS^•+^ and DPPH^•^ in whole fruit (W) between citrus cultivars. ^1,2^Legend: see Table 1.

**Table 5:** Content of heavy metals (*μ*g/100 g) in citrus pulp (F), peel (P), and whole fruits (W).

Fruit/index	Part	Orange	Pomelo	Mandarin	Lemon	Key lime	Red grapefruit	Green grapefruit	White grapefruit
Lead	F	1.00 ± 0.039^d^	1.67 ± 0.059^c^	0.354 ± 0.031^e^	1.67 ± 0.018^c^	1.91 ± 0.120^b^	2.47 ± 0.132^a^	1.83 ± 0.150^b^	1.01 ± 0.081^d^
	P	1.02 ± 0.046^e^	2.96 ± 0.195^a^	2.02 ± 0.157^d^	1.88 ± 0.062^de^	2.65 ± 0.104^b^	3.01 ± 0.094^a^	2.07 ± 0.135^d^	2.28 ± 0.115^c^
	W	1.00 ± 0.037^f^	2.18 ± 0.222^bc^	1.10 ± 0.135^f^	1.75 ± 0.099^de^	2.25 ± 0.213^b^	2.72 ± 0.247^a^	1.96 ± 0.197^cd^	1.59 ± 0.099^e^

Cadmium	F	0.015 ± 0.002^d^	0.039 ± 0.007^b^	0.017 ± 0.002^d^	0.022 ± 0.002^cd^	0.004 ± 0.001^e^	0.015 ± 0.001^d^	0.025 ± 0.002^c^	0.116 ± 0.012^a^
	P	0.049 ± 0.003^c^	0.136 ± 0.015^b^	0.062 ± 0.003^c^	0.047 ± 0.002^c^	0.046 ± 0.002^c^	0.191 ± 0.012^a^	0.199 ± 0.026^a^	0.137 ± 0.013^b^
	W	0.034 ± 0.005^de^	0.099 ± 0.010^bc^	0.043 ± 0.009^d^	0.038 ± 0.004^de^	0.023 ± 0.005^e^	0.090 ± 0.004^c^	0.110 ± 0.009^b^	0.131 ± 0.030^a^

Statistical analysis	*p*_value_								

F^1^*vs.* P									

Lead		0.422	<0.001	<0.001	<0.001	<0.001	<0.001	0.003	<0.001
Cadmium		<0.001	<0.001	<0.001	<0.001	<0.001	<0.001	<0.001	0.002

F^2^*vs.* W									

Lead		0.805	<0.001	<0.001	0.036	0.001	0.022	0.152	<0.001
Cadmium		0.004	<0.001	<0.001	<0.001	<0.001	<0.001	<0.001	0.213

^a,b,c,d,e,f^Values in rows with different letters are significantly different at *p* ≤ 0.05: F rows—superscript letters indicate statistically significant differences in heavy metal content in the pulp (F) between citrus cultivars; P rows—superscript letters indicate statistically significant differences in heavy metal content in the peel (P) between citrus cultivars. The results of two-way analysis of variance (ANOVA). W rows—superscript letters indicate statistically significant differences in heavy metal content in whole fruit (W) between citrus cultivars. ^1,2^Legend: see Table 1.

**Table 6:** Correlation coefficients between ADPH^•+^ or DPPH^•^ and content of phenolic compounds, ascorbic acid, and tannins for citrus pulp (F), peel (P), and whole fruits (W).

Fruit/index	Part	Orange	Pomelo	Mandarin	Lemon	Key lime	Red grapefruit	Green grapefruit	White grapefruit
Phenolic acids × ABTS^•+^	F	0.158	-0.358	0.235	-0.163	0.478	0.241	-0.074	-0.326
	P	0.063	0.680^∗^	0.176	-0.273	-0.009	0.019	0.460	-0.614
	W	0.265	0.052	0.190	-0.029	0.488	−0.691^∗^	-0.086	0.287

Tannins × ABTS^•+^	F	-0.082	0.179	0.371	-0.427	0.240	-0.473	0.021	-0.030
	P	-0.584	-0.398	-0.489	0.483	-0.204	0.329	-0.704	-0.092
	W	-0.147	0.228	-0.177	0.113	0.038	-0.549	-0.013	-0.319

Ascorbic acid × ABTS^•+^	F	0.485	0.038	0.013	-0.216	-0.470	-0.132	0.485	0.079
	P	-0.021	0.672^∗^	0.519	-0.257	-0.031	0.236	0.183	-0.243
	W	0.413	0.694^∗^	-0.089	-0.424	-0.026	−0.643^∗^	0.245	0.218

Phenolic acids × DPPH^•^	F	-0.719	0.429	0.632^∗^	0.241	-0.359	-0.104	-0.183	-0.448
	P	0.172	0.127	0.137	0.080	0.409	0.422	-0.378	0.144
	W	0.176	-0.308	-0.509	0.274	-0.509	-0.553	-0.354	0.048

Tannins × DPPH^•^	F	−0.697^∗^	-0.401	0.535	0.432	0.311	0.660^∗^	-0.057	-0.009
	P	0.442	-0.242	-0.324	0.405	0.670^∗^	-0.187	-0.500	0.328
	W	-0.566	−0.640^∗^	-0.051	-0.461	-0.147	−0.862^∗^	-0.378	0.026

Ascorbic acid × DPPH^•^	F	-0.184	0.738^∗^	-0.382	-0.082	-0.381	-0.618	0.270	-0.499
	P	0.717^∗^	-0.017	0.884^∗∗^	0.043	0.768^∗^	0.222	0.440	-0.105
	W	0.772^∗^	-0.473	-0.251	-0.146	-0.120	−0.809^∗^	-0.155	-0.595

^∗^Correlation is significant at *p* < 0.05. ^∗∗^Correlation is significant at *p* < 0.01.

## Data Availability

Partial data can be found with the authors in Excel.

## Additional Points

*Highlight*. The peel of citrus fruits has less glucose and fructose contents than the pulp. The peel of citrus is richer in fibre, tannins, and phenolic components than the pulp. Whole citrus fruits, and not just the pulp, may increase health benefits. A significant proportion of the bioactive compounds are found in the citrus peel. The cadmium and lead contents of the whole citrus fruit were below the ADI.
